# The kidney: function, cells and biomarkers

**DOI:** 10.1590/2175-8239-JBN-2020-0215

**Published:** 2021-01-06

**Authors:** Maurício Younes-Ibrahim

**Affiliations:** 1 Universidade do Estado do Rio de Janeiro, Rio de Janeiro, RJ, Brasil.; 2 Pontifícia Universidade Católica do Rio de Janeiro, Rio de Janeiro, RJ, Brasil.

 "Experience Teaches"

*Claude Bernard*

The kidney plays a key role in homeostasis, maintains the internal environment and ensures the physiological environment for approximately 100 trillion cells in the human body. The nephron's anatomic histological structure, with more than 20 types of specialized cells, reveals an architecture strategically focused on the impeccable endothelium/epithelium functional interaction. Pathophysiological conditions can cause changes to this internal environment and to the urine, evidenced by markers of dysfunction and molecules involved with cell injury. Uremia was the first biomarker of Kidney dysfunction (18th century), and became a synonym for kidney failure (KF). Creatinine (19th century) brought a better understanding of glomerular filtration and, since then, it is the most used biomarker in nephrology. Cystatin C (20th century) is the most recent biomarker established to quantify and classify the stages of kidney function. These molecules, in high concentrations, integrate the classical KF metabolome, and represent the metabolic signature of different past biological phenomena which, in an acute or chronic way, produced kidney dysfunction.

With a range of elements, urinalysis currently has a set of physiological and pathophysiological biomarkers, capable of revealing informations set in three moments in time: biological phenomena that have already occurred, phenomena with occurrences in the present, or even predictive of future events, located or not in the urinary pathways. Taking urinary proteins for instance, we can find them in each of these moments with, respectively, myoglobinuria, Bence Jones protein and microalbuminuria.

Despite being low cost and easy to dose, creatinine has a serum half-life of 4 hours and takes up to 40 hours for its concentration to reveal Acute Kidney Injury (AKI) a long time when it comes to critically ill patients. AKI's epidemiological growth of high mortality in the XXI century triggered the search for early diagnosis of tubular epithelial cell disease,^1^ especially acute tubular necrosis (ATN), aiming at anticipating the necessary therapeutic measures and to intervene favorably in the natural history of AKI. Thus, several new markers have been proposed:^2^ NGAL (neutrophyl gelatinase-associated lipocalin); KIM-1 (Acute Kidney Injury molecule-1); L-FABP (liver fatty acid-binding protein); IGFBP7 (insulin-like growth factor-binding protein 7); IL-18 (interleukin-18); TIMP-2 (metallopeptidase-2 inhibitor); MCP-1 (monocyte chemotactic peptide-1); CCL-14 (chemokine CCL-14); CHI3L1 (chitinase-3-like protein 1).

As Claude Bernard indoctrinated in the Principles of Experimental Medicine (1865),^3^ "Experience Teaches": when phenomena are constantly put to test, they confirm their hypotheses or not. In this sense, since their discoveries, several studies have tested different ATN biomarkers, aiming to validate them in the diversity of clinical practice. As they reveal damage to the tubular epithelium, some of these parameters suffer interference from age, sex, urinary infection and chronic kidney disease, especially in tubule-interstitial involvement, which somehow affects the epithelial cells.^4^

In this edition, the relevant study by Tavares et al^5^ shows a sensible clinical evaluation strategy using two acute tubular lesion markers, NGAL and KIM-1, performed on patients who had sudden loss of kidney function, but who were known to have nephrotic syndrome disease of varied causes. The recent development of specific biomarkers for the characterization of glomerular diseases has gradually been filling gaps in the knowledge of glomerulopathies.^6^ However, in the clinical course of nephrotic syndrome it is not uncommon for patients to present acute elevation of serum creatinine due to toxic, ischemic or NTA-origin sepsis. The diagnostic need is due to the fact that acute dysfunction could also result from a worsening of the underlying glomerular disease, sometimes requiring kidney biopsy for clarification. The importance of this definition is justified, since necessary therapeutic interventions are diverse, according to the event diagnosed. In this series, the authors describe that simultaneous use of the two urinary biomarkers identified a positive and effective histopathological association to differentiate whether the appearance of acute kidney dysfunction resulted from overlap of an epithelial insult or from intensification of glomerular insult. This contribution shows a potential practical usefulness of this method to monitor patients with different forms of glomerular diseases.

With this publication, Tavares et al. stimulate new investigations on the subject to be done with NGAL, KIM-1 and the other molecules. Second generation biomarkers, TIMP-2 and IGFBP-7,^7^ which reflect the cell-cycle interruption of cells in the proximal and distal tubular epithelium, will probably be tested. Some of them detect cell insults,^7^^,^ 8 including sublethal ones, from clinical or surgical stress, even in the first 12 hours, as they are also used to identify the persistence of AKI epithelial lesion in AKIN 2 and 3 stages.^9^ The promising expectation is that, in the near future, these biomarkers will consolidate as tools for risk stratification and severity in AKI, as well as for the definition of personalized clinical procedures, including in glomerulopathies.

Therefore, nephrology also teaches us that the inseparable endothelium-epithelium interconnection, so necessary for physiology, is not undone by pathophysiological circumstances, in which, effectively, glomerulopathies are not dissociated from tubulopathies.
